# Survival, treatment regimens and medical costs of women newly diagnosed with metastatic triple-negative breast cancer

**DOI:** 10.1038/s41598-021-04316-2

**Published:** 2022-01-14

**Authors:** Ju-Yi Hsu, Chee-Jen Chang, Jur-Shan Cheng

**Affiliations:** 1 Department of Biomedical Sciences, College of Medicine, Chang Gung University, Taoyuan, Taiwan; 2 Biotechnology Industry, College of Medicine, Chang Gung University, Taoyuan, Taiwan; 3 Clinical Informatics and Medical Statistics Research Center, College of Medicine, Chang Gung University, Taoyuan, Taiwan; 4 Research Services Center for Health Information, Chang Gung University, Taoyuan, Taiwan; 5 Graduate Institute of Clinical Medical Sciences, College of Medicine, Chang Gung University, Taoyuan, Taiwan; 6 Department of Obstetrics and Gynecology, Chang Gung Memorial Hospital, Taoyuan, Taiwan; 7 Department of Emergency Medicine, Chang Gung Memorial Hospital, Keelung, Taiwan

**Keywords:** Cancer, Health care

## Abstract

Individuals diagnosed with metastatic triple-negative breast cancer (mTNBC) suffer worse survival rates than their metastatic non-TNBC counterparts. There is little information on survival, treatment patterns, and medical costs of mTNBC patients in Asia. Therefore, this study aimed to examine 5-year survival, regimens of first-line systemic therapy, and healthcare costs of mTNBC patients in Taiwan. Adult females newly diagnosed with TNBC and non-TNBC as well as their survival data, treatment regimens and costs of health services were identified and retrieved from the Cancer Registry database, Death Registry database, and National Health Insurance (NHI) claims database. A total of 9691 (19.27%) women were identified as TNBC among overall BC. The 5-year overall survival rate of TNBC and non-TNBC was 81.28% and 86.50%, respectively, and that of mTNBC and metastatic non-TNBC was 10.81% and 33.46%, respectively. The majority of mTNBC patients received combination therapy as their first-line treatment (78.14%). The 5-year total cost in patients with metastatic non-TNBC and with mTNBC was NTD1,808,693 and NTD803,445, respectively. Higher CCI scores were associated with an increased risk of death and lower probability of receiving combination chemotherapy. Older age was associated with lower 5-year medical costs. In sum, mTNBC patients suffered from poorer survival and incurred lower medical costs than their metastatic non-TNBC counterparts. Future research will be needed when there are more treatment options available for mTNBC patients.

## Introduction

Triple-negative breast cancer (TNBC) is characterized by estrogen receptor-negativity (ER-), progesterone receptor-negativity (PR-) and human epidermal growth factor receptor 2-negativity (HER2-negative). Patients with TNBC account for 15–20% of all breast cancer patients in the US^1–3^. TNBC is considered to be an aggressive and difficult-to-treat cancer^4^. TNBC patients suffer the worst prognosis among all breast cancer types, and the overall survival is worse than non-TNBC patients^1,5–7^. In America, the 5-year overall survival rate is 77% in TNBC patients, compared with 93% in breast cancer patients of other types^1^. In German, the 5-year overall survival rate was 75.8% and 84.8% in TNBC and non-TNBC patients, respectively^8^. The 5-year overall survival rate in metastatic triple-negative breast cancer (mTNBC) patients was 4–20% in western countries^3,9–13^. In Taiwan, patients with TNBC account for 15–20% of all breast cancer patients^14–16^, which is similar to findings in western countries^1–3^. A study conducted in Taiwan demonstrated that both 10-year disease-free survival (DFS) and 10-year breast cancer-specific survival (BCSS) of patients with non-TNBC are higher than those of patients with TNBC^15^. So far, there have been few studies examining overall survival of TNBC patients by stage and by using representative samples to reflect survival of TNBC patients with Asian ethnicity.

Current treatments for mTNBC patients, however, are limited, compared with non-TNBC patients with expression of ER, PR and/or HER2. According to the National Comprehensive Cancer Network (NCCN) guidelines 2020^17^, the recommended treatment for the mTNBC patients is single drug treatment. Combined chemotherapy is for patients with a high tumor burden, rapid disease deterioration, and visceral crisis of organ metastasis. The most recommended treatment options for mTNBC patients are doxorubicin, liposomal doxorubicin, paclitaxel, capecitabine, gemcitabine, vinorelbine, eribulin, olaparib, talazoparib, cisplatin, carboplatin and atezolizumab combined with nab-paclitaxel. For some circumstances, the recommended treatment options were combination treatments such as doxorubicin/cyclophosphamide (AC), epirubicin/cyclophosphamide (EC), cyclophosphamide/methotrexate/fluorouracil (CMF), docetaxel/capecitabine, and paclitaxel/bevacizumab. Previous studies found that 65–67% of mTNBC patients in the US^9^, Canada^10^ and Germany^18^ received monotherapy as their first-line treatment rather than combination regimens. In the US, 39% of them received taxane monotherapy. However, in Asian country such as Thailand, 65% of mTNBC patients received combination therapy^19^. In Taiwan, the current treatment options as first-line systemic therapy for mTNBC patients available in the National Health Insurance (NHI) program are docetaxel, doxorubicin, liposomal doxorubicin, vinorelbine, uracil-tegafur, capecitabine, cisplatin, and carboplatin. However, the treatment regimens of first-line systemic therapy and factors associated with choice of treatment need further examination.

Healthcare costs of mTNBC patients have been examined in western countries^9,20,21^. Of the elderly mTNBC patients in the US, the mean cumulative cost was US$73,586, and mean cost in first-line and second-line treatments was US$26,950 and US$33,347, respectively. Physician/clinic cost was the highest during pre-treatment, first regimen, second regimen, and third and subsequent regimens phases^9^. Another study also examined costs during different lines of systemic therapy in mTNBC patients in the US and found that the mean total cost increased from US$21,908 in the first-line therapy to US$25,845 in the third-line therapy^20^. Hospitalization costs accounted for almost half of the total cost, followed by the costs of systemic anti-cancer therapy. In Canada, the mean annual per-patient cost of adult stage IV TNBC patients was $140,160, with inpatient services accounted for 45.4% of the total cost^21^. As far as we know, there is no study examining medical costs of mTNBC patients in Taiwan.

There is little information on survival, treatment regimens, and medical costs of TNBC patients in Asia. Therefore, this study aimed to examine 5-year survival, regimens of first-line systemic chemotherapy, and healthcare costs of mTNBC patients in an Asian country through a retrospective cohort study design by using nation-level databases.

## Methods

### Data and sample

This was a population-based observational study using nation-level data, including the Cancer Registry Database, the Death Registry Database, and the National Health Insurance (NHI) administrative database. In Taiwan, the single-payer, mandatory NHI program provides a comprehensive benefits package, including outpatient and inpatient services, laboratory tests, and prescription drugs^22^. Nearly 99% of the Taiwanese population are enrolled in the NHI program, and more than 92% of health care organizations had contracts with the National Health Insurance Administration (NHIA).

In this study, female subjects aged at least 18 years old newly diagnosed with breast cancer during 2008 and 2013 were selected from the Cancer Registry data (Supplementary Fig. 1). They were further divided into a TNBC group and a non-TNBC group, based on treatment they received during the study period. Those who had ever received hormone therapy or HER2 therapy were defined as non-TNBC. Survival of the TNBC group and non-TNBC group, overall and by stage, was investigated. Cancer stage at diagnosis was based on AJCC staging system adopted in the Cancer Registry database. Those with stage IV TNBC (de novo metastatic TNBC) were further examined for their overall survival, first-line treatment regimens, and medical costs during the 5-year follow-up by age and lymph node status.

### Measurements

Overall survival was the time from the date of diagnosis (the index date) until death or the end of 5-year follow-up, whichever came first. Use and costs of health services were retrieved from the NHI administrative database. First-line chemotherapy regimens for mTNBC patients included doxorubicin, liposomal doxorubicin, paclitaxel, capecitabine, gemcitabine, vinorelbine, eribulin, cisplatin, and carboplatin that were reimbursed by the NHI program. Costs were divided into cancer-related and cancer-unrelated inpatient and outpatient costs based on the diagnosis codes and settings of care delivery. Costs were expressed in New Taiwan Dollars (NTD). The conversion rate of NTD to US dollars (USD) was approximately 30:1.

### Statistical approaches

Continuous variables between the non-TNBC and TNBC groups were compared with t-tests. Chi-square tests were performed to compare categorical variables between the non-TNBC and TNBC groups. The Kaplan–Meier method was used to estimate the overall survival. The differences in cumulative survival between the non-TNBC and TNBC groups were examined by the log-rank test. The Cox proportional hazard regression models were adopted to examine factors associated with overall survival. To investigate factors associated with the choice of single-drug chemotherapy or combination therapy, a logistic regression model was used. Factors associated with 5-year medical costs were examined by adopting a generalized linear model with a log link and gamma distribution. Covariates in the models include TNBC (yes, no), age (< 40, 40–59, ≥ 60), lymph node status (positive, negative), and Charlson comorbidity index (CCI) score^23^ (0, 1–2, > 2). A *p* value of < 0.05 was considered statistically significant. All statistical analyses were performed using SAS 9.4 software (SAS Institute, Inc., Cary, NC, USA).

This study was granted ethical approval by the Institutional Review Board of the Chang Gung Memorial Hospital of Taiwan. Informed consent was waived because personal identification numbers were encrypted in the data.

## Results

### Characteristics of the sample

Of 50,856 subjects identified in this study, 9691 were TNBC (19.27%) and the remaining 40,589 were non-TNBC (80.73%) (Supplementary Table 1). The mean ages of the TNBC group and non-TNBC group were 54.21 and 53.62, respectively. TNBC patients accounted for 20.34%, 20.80%, 15.24%, and 15.65% of stage I, stage II, stage III and metastatic BC patients, respectively. The rate of positive lymph node status in non-TNBC group and TNBC group was 48.76% and 36.61%, respectively.

Of de novo metastatic breast cancer (MBC) subjects, there was no significant difference in distributions of age and known lymph node status between mTNBC (N = 536, 15.65%) and metastatic non-TNBC (N = 2888, 84.35%) (Table 1). Subjects with mTNBC had higher CCI score than those with metastatic non-TNBC.

**Table 1:** Characteristics of the subjects with metastatic breast cancer.

	Non-TNBC		TNBC		*p* value
	N	%	N	%	
Total	2888	84.35	536	15.65	
**Age**					0.2305
< 40	219	7.58	40	7.46	
40–59	1717	59.45	317	59.14	
≥ 60	952	32.96	179	33.40	
**Lymph node status**					0.0104
Negative	238	8.24	52	9.70	0.1705^a^
Positive	2450	84.83	429	80.04	
Unknown	200	6.93	55	10.26	
**CCI score**					0.0429
0	1802	62.40	318	59.33	
1–2	486	16.83	81	15.11	
> 2	600	20.78	137	25.56	

CCI: Charlson comorbidity index; SD: standard deviation.

^a^Test for known lymph node status only.

### Overall survival of TNBC group vs. non-TNBC group

**Figure 1:**
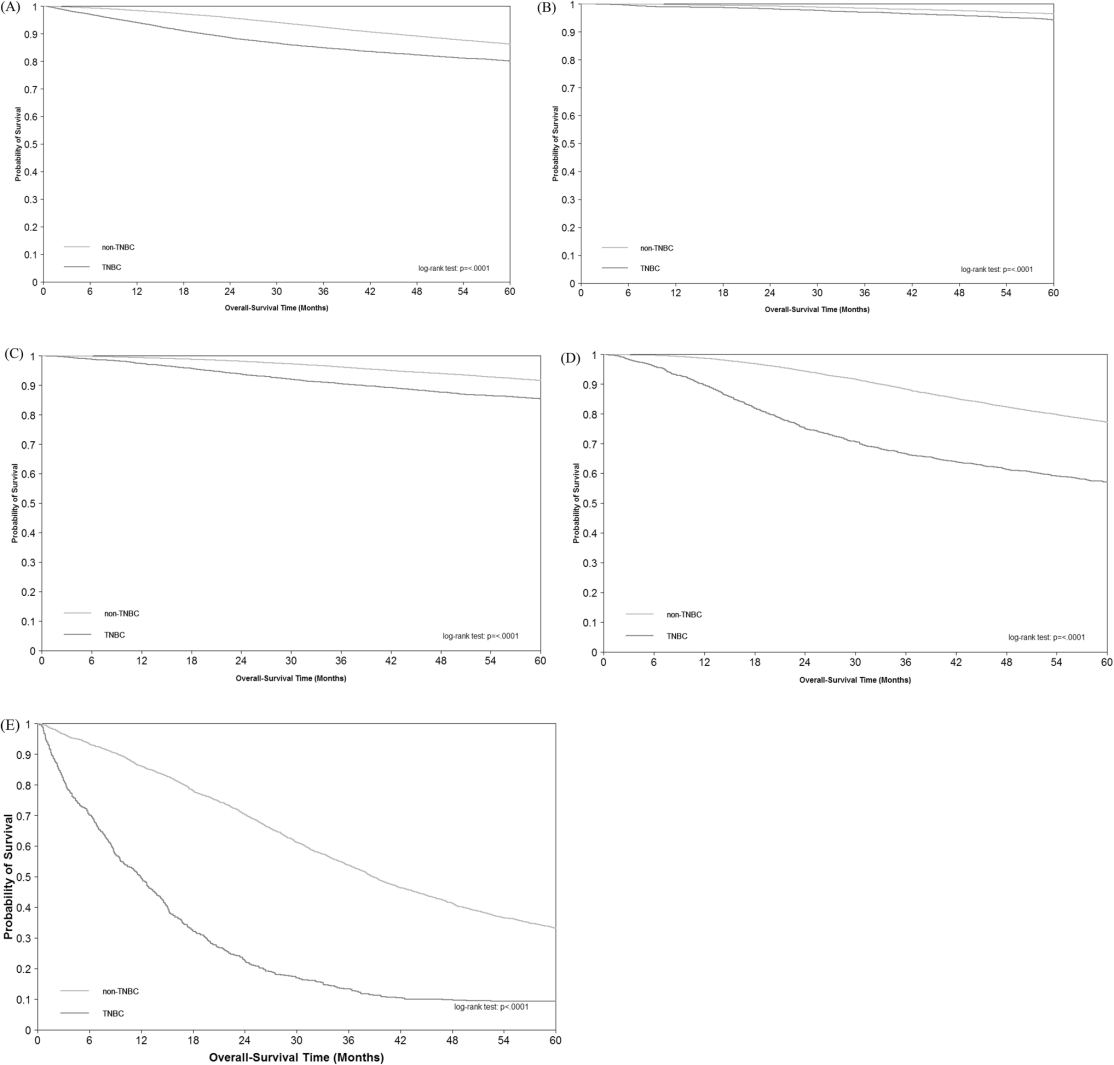
Overall survival of TNBC vs. non-TNBC patients by stage: (**A**) overall; (**B**) stage I; (**C**) stage II; (**D**) stage III; (**E**) stage IV.

**Figure 2:**
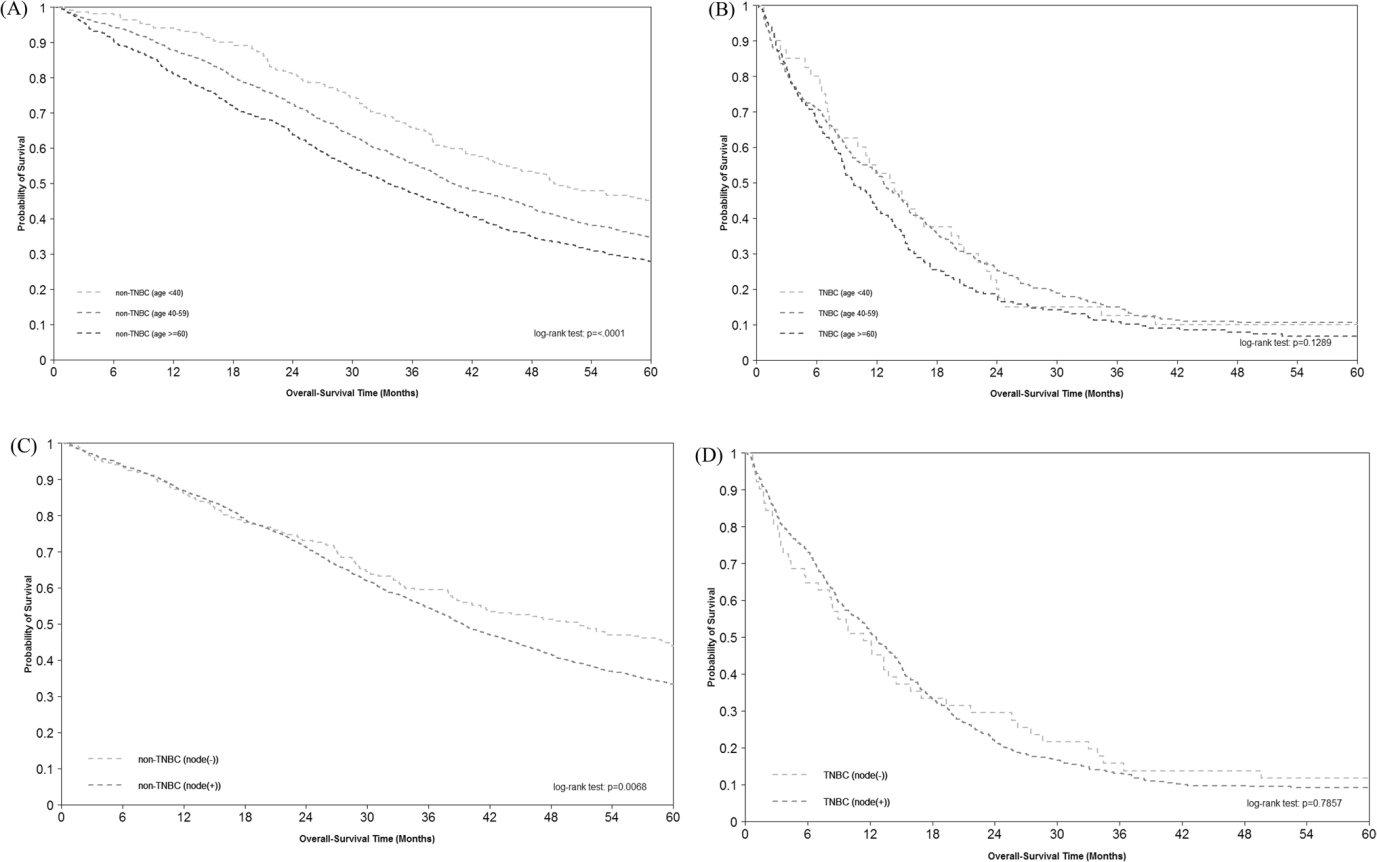
Overall Survival of MBC: (**A**) non-TNBC by age; (**B**) TNBC by age; (**C**) non-TNBC by lymph node status; (D) TNBC by lymph node status.

The 5-year overall survival in non-TNBC and TNBC patients was shown in Fig. 1. The 5-year overall survival rate in non-TNBC and TNBC patients were 86.50% and 81.28%, respectively (Fig. 1A). The 5-year overall survival rate of TNBC group of stage I, stage II, stage III, and stage IV was 94.74%, 86.38%, 59.27%, and 10.81%, respectively (Fig. 1B–E). In non-TNBC group, the 5-year overall survival rate of stage I, stage II, stage III, and stage IV was 96.50%, 91.84%, 77.48%, and 33.46%, respectively (Fig. 1B–E). The overall survival among TNBC and non-TNBC groups differed significantly overall and in each stage, with the TNBC group suffering from poorer overall survival compared with the non-TNBC group, particularly in stage IV BC (Fig. 1E). Subjects with mTNBC, compared with subjects with metastatic non-TNBC, had worse overall survival in different age groups (Supplementary Fig. 2) and lymph node status (Supplementary Fig. 3). Overall survival rates of different age groups and lymph node status differed in the non-TNBC group but not in the TNBC group (Fig. 2). For patients with mTNBC, the 5-year overall survival rate of age < 40, age 40–59 and age ≥ 60 was 10.00%, 11.36%, 7.82%, respectively. That for patients with non-TNBC was 45.21%, 34.83%, 28.27, respectively. In mTNBC group, the 5-year overall survival for lymph node-positive status and lymph node-negative status was 13.46% and 9.75%, respectively. In comparison the survival rate in the metastatic non-TNBC group was 44.54% and 33.63%, respectively.

In patients with MBC, TNBC (HR = 3.33, 95% CI: 2.99–3.71), older age (HR = 1.42, 95% CI: 1.19–1.70), lymph node-positive status (HR = 1.32, 95% CI: 1.13–1.54), and higher CCI scores (HR = 1.63, 95% CI: 1.47–1.81) were associated with increased risk of death (Table 2). In the subgroup of mTNBC, a higher CCI score (HR = 1.46, 95% CI: 1.16–1.83) was associated with higher probability of death.

**Table 2:** Factors associated with risk of death, use of combination chemotherapy, and 5-year medical costs.

Variable	Risk of death^a^				Combination chemotherapy^b^		Medical cost^c^			
	MBC		mTNBC		mTNBC		MBC		mTNBC	
	HR (95% CI)	*p* value	HR (95% CI)	*p* value	OR (95% CI)	*p* value	Estimate (SE)	*p* value	Estimate (SE)	*p* value
**TNBC (reference: non-TNBC)**										
Yes	3.33 (2.99–3.71)	< .0001					− 0.80 (0.04)	< .0001		
**Age (reference: < 40)**										
40–59	1.17 (0.99–1.39)	0.0706	0.85 (0.59–1.22)	0.3773	1.27 (0.49–3.28)	0.6270	− 0.08 (0.05)	0.1205	− 0.04 (0.14)	0.7900
60	1.42 (1.19–1.70)	0.0001	0.97 (0.66–1.43)	0.8849	0.53 (0.20–1.42)	0.2072	− 0.34 (0.05)	< .0001	− 0.40 (0.14)	0.0058
**Lymph node status (reference: negative)**										
Positive	1.32 (1.13–1.54)	0.0004	1.10 (0.80–1.51)	0.5543	0.69 (0.28–1.67)	0.4081	0.14 (0.04)	0.0014	0.11 (0.11)	0.3265
**CCI score (reference: CCI score = 0)**										
1–2	1.09 (0.97–1.23)	0.1514	1.16 (0.87–1.55)	0.3241	0.70 (0.35–1.38)	0.3051	− 0.09 (0.04)	0.0141	0.07 (0.11)	0.5063
> 2	1.63 (1.47–1.81)	< .0001	1.46 (1.16–1.83)	0.0014	0.53 (0.30–0.92)	0.0252	− 0.12 (0.03)	0.0004	− 0.03 (0.09)	0.7183

^a^Cox proportional hazard model.

^b^Logistic regression model.

^c^Generalized linear model.

### First-line systemic chemotherapy regimens of mTNBC

The treatment regimens of first-line chemotherapy were shown in Supplementary Table 2. The majority of mTNBC patients received combination chemotherapy as their first-line treatment (78.14%). Platinum + taxane was the most common option for dual combination treatment (9.96%), while cyclophosphamide/doxorubicin/fluorouracil or cyclophosphamide/epirubicin/fluorouracil (CAF/CEF) was the most commonly used triple combination treatment (27.06%). Taxanes were the most commonly used first-line single chemotherapy (11.26%). Older mTNBC patients tended to use monotherapy (33.57%) more than younger mTNBC patients (16.67%) (Table 3), while choices of treatments were not different in different lymph node status. A higher CCI score (< 2) (OR = 0.53, 95% CI: 0.30–0.92) was associated with a reduced likelihood of receiving combination therapy (Table 2).

**Table 3:** First-line chemotherapy treatment regimens in mTNBC patients by age and lymph node status.

	Age < 40 N = 36	Age 40–59 N = 283	Age ≥ 60 N = 143	*p* value	Node (−) N = 38	Node (+) N = 388	*p* value
**Regimens (%)**				0.0002			0.6431
Monotherapy	16.67	16.61	33.57		18.42	21.65	
Combination therapy	83.33	83.39	66.43		81.52	78.35	
**Number of agents (%)**				0.0002			0.7391
Single agents	16.67	16.61	33.57		18.42	21.65	
Dual agents	41.67	33.57	34.97		42.11	34.28	
Triple agents	30.56	45.94	28.67		34.21	40.21	
More agents	11.11	3.89	2.80		5.26	3.87	

### Costs of health services of mTNBC

**Figure 3:**
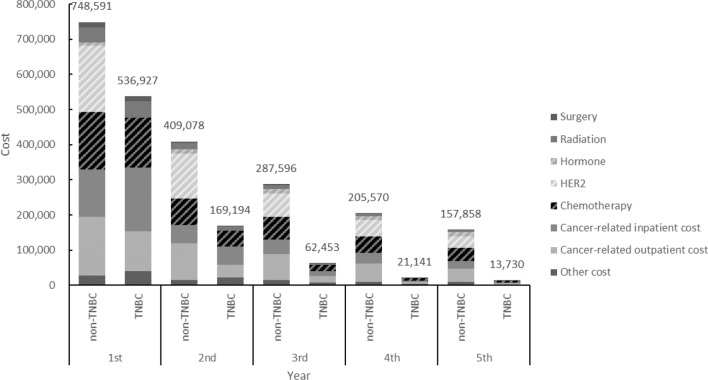
Mean medical cost per patient per year after diagnosis with metastatic non-TNBC or mTNBC.

The 5-year total cost in patients with metastatic non-TNBC was significantly higher than patients with mTNBC (NTD1,808,693 vs. NTD803,445) (Table 4). The pattern was also found in different age groups and lymph node status. In both groups, annual total cost was the highest in the first year after diagnosis, and decreased afterwards (Fig. 3). In the first 2 years, majority of medical costs was due to chemotherapy and hospitalization in mTNBC patients. In the metastatic non-TNBC group, the cost of HER2 and hormone therapy was the majority cost during the 5-year follow-up period. Similar patterns were found in different age groups and lymph node status (Supplementary Fig. 4(A) and (B)). Older patients incurred lower medical costs in both groups, while subjects with positive lymph node status incurred higher medical costs in metastatic non-TNBC group. In individuals with MBC, TNBC, older age, and a higher CCI score were associated with lower medical costs, while positive lymph node status was correlated with higher medical cost (Table 2). In the subgroup of mTNBC, older age was associated with increased medical costs.

**Table 4:** Five-year total medical cost in subjects with metastatic non-TNBC and mTNBC.

	Non-TNBC		*p* value	TNBC		*p* value	Non-TNBC vs. TNBC
	Mean	SD		Mean	SD		*p* value
Total cost	1,808,693	1,305,213		803,445	646,201		< .0001
**Age**			< .0001			< .0001	
< 40	2,139,121	1,329,958		1,003,698	751,239		< .0001
40–59	1,954,559	1,340,796		874,604	659,375		< .0001
60	1,469,603	1,159,166		632,677	558,766		< .0001
**Lymph node status**			0.0001			0.1797	
Negative	1,560,142	1,217,055		711,355	581,689		< .0001
Positive	1,858,400	1,312,060		822,915	646,866		< .0001

## Discussion

In this study, TNBC accounted for 19.27% of breast cancer patients, ranging from 15.24% to 20.80% in stage I–IV. The TNBC group had poorer overall survival compared with the non-TNBC group overall, and by stage, age, and lymph node status., The majority of mTNBC patients received combination chemotherapy as their first-line treatment, with CAF/CEF the most commonly used chemotherapy. Mean 5-year total medical costs of the metastatic non-TNBC group was higher than that of the TNBC group, and the annual cost decreased after the first year after diagnosis in both groups. Higher CCI scores were associated with an increased risk of death and lower probability of receiving combination chemotherapy as first-line therapy. Older age was associated with lower 5-year medical costs.

This study demonstrated that TNBC accounted for 20.34%, 20.80%, 15.24%, 15.65% of stage I, stage II, stage III and stage IV patients, respectively. The proportion of TNBC among BC patients was higher in early stages than in later stages. The findings were consistent with previous studies^1–3,14–16^.

The 5-year overall survival rates of the non-TNBC group were significantly higher than those of the TNBC group overall (86.50% and 81.28%), and in stage I (96.50% and 94.74%), stage II (91.84% and 86.38%), stage III (77.48% and 59.27%), and stage IV (33.46% and 10.81%). These findings were consistent with previous studies^1,5–8,15,24^. The survival rate of mTNBC (10.81%) was comparable to those found in other countries, ranging from 4% to 20%^3,9–13^.

The findings of this study indicated that having severe comorbid conditions (higher CCI scores) significantly increased the risk of mortality in mTNBC, consistent with previous studies that took into account the physical condition in patients with mTNBC^24^ or MBC^25^. In this study, it was demonstrated that older age and positive lymph node status increased the risk of death in MBC but not in mTNBC. Older age was found to be associated with a higher risk of death in MBC^6,11^, but the association in mTNBC was inconclusive^11,24^. The correlation between lymph node status and survival in MBC was mixed^6,11^.

In this study, 90.10% mTNBC patients received chemotherapy. We also found that most mTNBC patients received combination chemotherapy (78.14%) as their first-line treatment, comparable to findings in Taiwan^24^ and Thailand^19^ (76% and 65%, respectively). However, this is different from mTNBC patients in the US^9^, Canada^10^ and Germany^18^ where 65–67% received monotherapy as first-line treatment. Cyclophosphamide, anthracycline, and fluorouracil (CAF/CEF) was the most commonly used first-line treatment (27%) for mTNBC patients in this study, consistent with previous studies in Taiwan^24^ and in Thailand^19^ (24% and 32%, respectively). Of those receiving single-agent chemotherapy, 51% of them received taxane in this study, compared with only 39% in the US. Even though the preferences for first-line treatment regimens were different in Asian and western countries, we further demonstrated that combination chemotherapy was used more commonly in younger patients, and having severe comorbidities is a crucial concern in choosing single-agent over combination therapy.

In our study, it was found that the total cost from first year to fifth year gradually decreased and most of the costs were incurred by cancer-related health services in mTNBC patients. In addition, hospitalization accounted for a major proportion of medical costs as observed in previous studies^20,21^. These findings may reflect the treatment patterns of mTNBC patients. Once diagnosed, patients usually receive intensive treatments and then are monitored closely during follow-up. Even though average treatment costs increased from first-line therapy to therapies of subsequent lines^9,20^, the downward trend of mean total cost may be affected by decreasing number of patients receiving later lines of therapy. The findings of this study further demonstrated that older age was associated with lower medical costs. Compared with younger patients, the more elderly tended to incur lower cost of chemotherapy, which may partly reflect their first-line treatment regimens. However, further study is needed to identify more details in health service utilization so as to understand the disease burden due to different aspects of disease management such as subsequent lines of treatments, severe adverse events, disease monitoring, and palliative care.

During the study period, treatment options available for mTNBC patients under NHI coverage were chemotherapy only. Therefore, treatment choices and medical costs examined may not reflect current situations in mTNBC patients. There were two poly ADP ribose polymerase (PARP) inhibitors (olaparib and talazoparib) reimbursed by the NHI program for mTNBC patients with BRCA mutation in 2021. However, none of immune-check point inhibitors are available so far for mTNBC patients in the NHI program. Future research will be needed to examine treatment options and medical costs of mTNBC patients, particularly after adoption of immune-check point inhibitors which absolutely will lead to high NHI spending.

There were three limitations in this study. First, while status of ER, PR and HER-2 expression was not available in the Cancer Registry Database, the receipt of hormone therapy or HER2 therapy during study period was used to classify TNBC and non-TNBC patients. Therefore, we are unable to distinguish non-TNBC from TNBC patients who never received hormone therapy or HER2 therapy, and the number and proportion of TNBC subjects might be overestimated. However, the proportions of TNBC among BC patients, distributions of cancer stage and lymph node state, and their survival were similar to those found in previous studies^14–16,21^. Second, the sixth edition and seventh edition of the AJCC Staging Manual was adopted by the Cancer Registry system before and after 2010, respectively. Therefore, subjects classified as in the same stage may have different tumor and node classification. However, the major study subjects of stage IV BC were classified by the definition of distant metastasis which is relatively consistent between the two editions. Third, information on the use and costs of health services was retrieved from the NHI claims database. Therefore, medications and services not reimbursed by the NHI program as well as our identification of complexity of treatment patterns and estimates of costs may be underestimated.

## Conclusions

Patients with mTNBC, accounting for 15.65% among MBC patients, suffered from worse survival and incurred lower medical costs than their metastatic non-TNBC counterparts. The majority of first-line chemotherapy in patients with mTNBC was combination therapy. Severe comorbidities were associated with mortality and choice of first-line chemotherapy, and age was correlated with medical costs. Future research will be needed when more treatment options such as immune-check point inhibitors are available for mTNBC patients.

## Supplementary Information

Supplementary Information.
