# Supplementary material for: Survival, treatment regimens and medical costs of women newly diagnosed with metastatic triple-negative breast cancer
Source: Sci Rep. 2022 Jan 14;12:729. doi: 10.1038/s41598-021-04316-2 (PMC8760241; doi:10.1038/s41598-021-04316-2)

Supplementary Table 1. Characteristics of subjects newly diagnosed with breast cancer

|  | non-TNBC  (n=40,589) | | TNBC  (n=9,691) | | p-value |
| --- | --- | --- | --- | --- | --- |
| Age (mean, SD) | 53.62 | 11.88 | 54.21 | 11.88 | <.0001 |
| Cancer stage (n, %) |  |  |  |  | <.0001 |
| I | 14,332 | 35.31 | 3,660 | 37.77 |  |
| II | 15,631 | 38.51 | 4,104 | 42.35 |  |
| III | 7,738 | 19.06 | 1,391 | 14.35 |  |
| IV | 2,888 | 7.12 | 536 | 5.53 |  |
| Lymph node status (n, %) |  |  |  |  | <.0001 |
| Negative | 20,554 | 50.64 | 6,014 | 62.06 | <.0001^a^ |
| Positive | 19,791 | 48.76 | 3,548 | 36.61 |  |
| Unknown | 244 | 0.60 | 129 | 1.33 |  |

TNBC, triple-negative breast cancer. SD, standard deviation.

^a^Test for known lymph node status only

Supplementary Table 2. First-line systemic treatment regimens of mTNBC

| Treatment | Number | % |
| --- | --- | --- |
| **Single Agent** |  |  |
| Paclitaxel | 29 | 6.28 |
| Docetaxel | 23 | 4.98 |
| Vinorelbine | 17 | 3.68 |
| Capecitabine | 15 | 3.25 |
| Tegafur | 5 | 1.08 |
| Cyclophosphamide | 3 | 0.65 |
| Anthracycline | 3 | 0.65 |
| Others | 6 | 1.30 |
| Subtotal | 101 | 21.86 |
| **Dual Combination** |  |  |
| Platinum+Taxane | 46 | 9.96 |
| AC/EC | 41 | 8.87 |
| GT | 19 | 4.11 |
| Taxane+Anthracycline | 17 | 3.68 |
| Fluorouracil+Vinorelbine | 7 | 1.52 |
| Platinum+Vinorelbine | 5 | 1.08 |
| Cyclophosphamide+Tegafur | 4 | 0.87 |
| Capecitabine+Taxane | 3 | 0.65 |
| Cyclophosphamide+Taxane | 3 | 0.65 |
| Others | 15 | 3.25 |
| Subtotal | 160 | 34.63 |
| **Triple Combination** |  |  |
| CAF/CEF | 125 | 27.06 |
| TAC/TEC | 40 | 8.66 |
| Others | 17 | 3.68 |
| Subtotal | 182 | 39.39 |
| **More Combination** | 19 | 4.11 |
| Total | 462 | 100.00 |

Note: A: doxorubicin; C: cyclophosphamide; E: epirubicin; F: fluorouracil; M: methotrexate; T: taxane

Supplementary Figure 1. Flow chart of subject selection


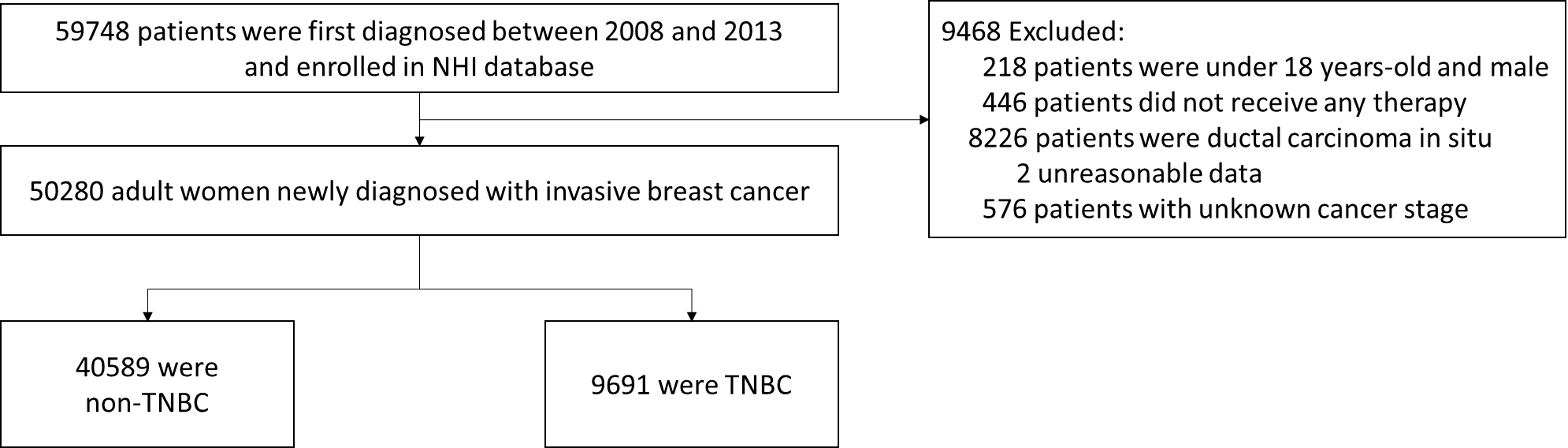


Supplementary Figure 2. Overall survival of MBC by age group: (A) age <40; (B) age 40-59; (C) age ≥ 60.

(A)


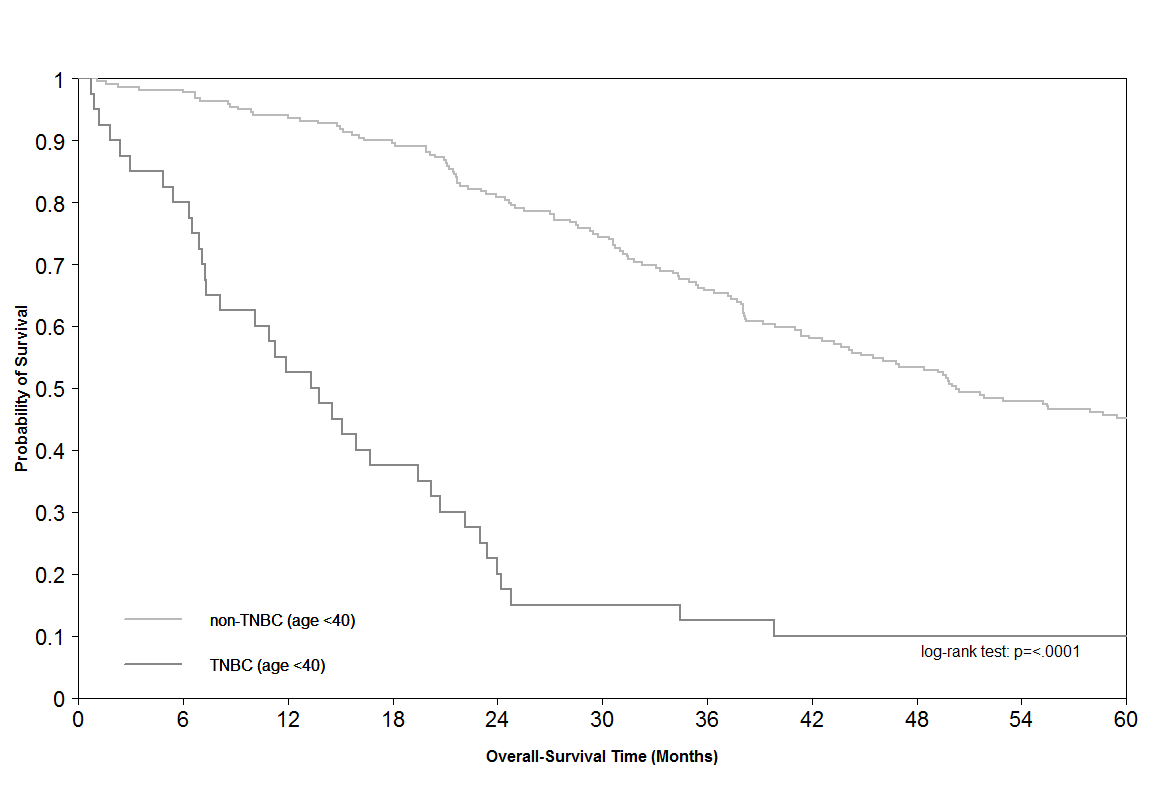


(B)


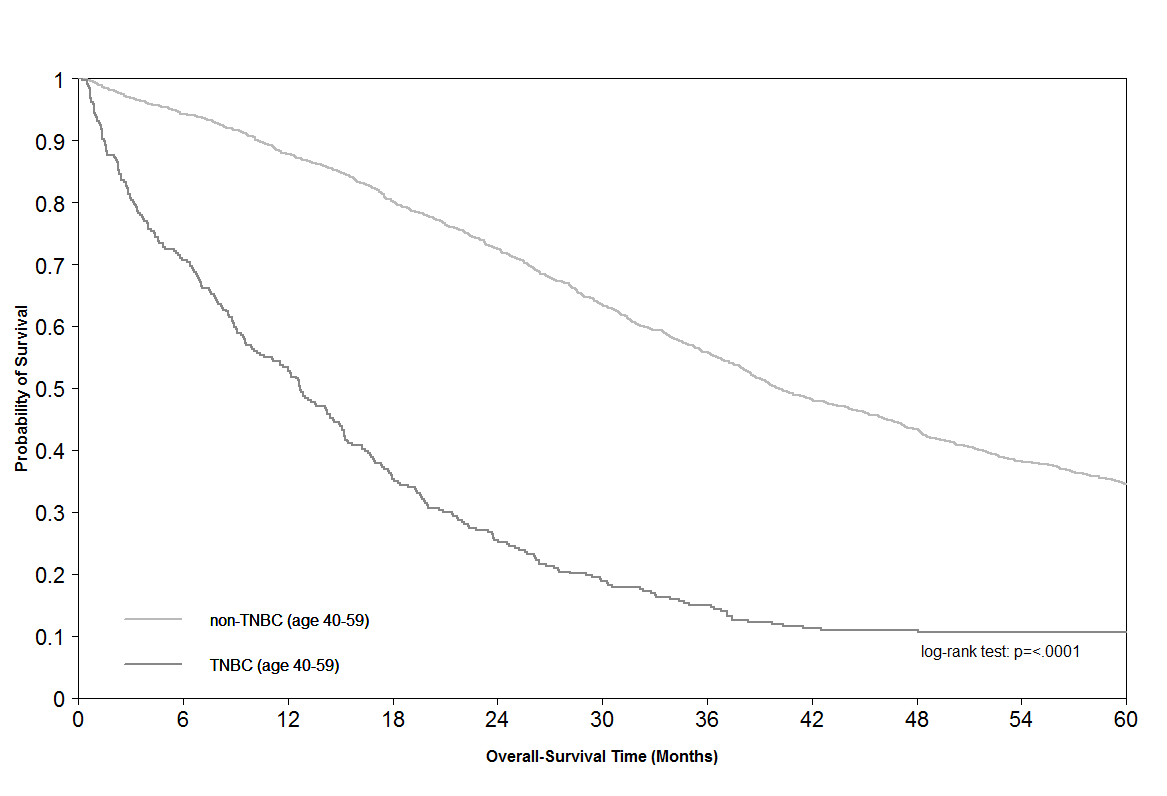


(C)


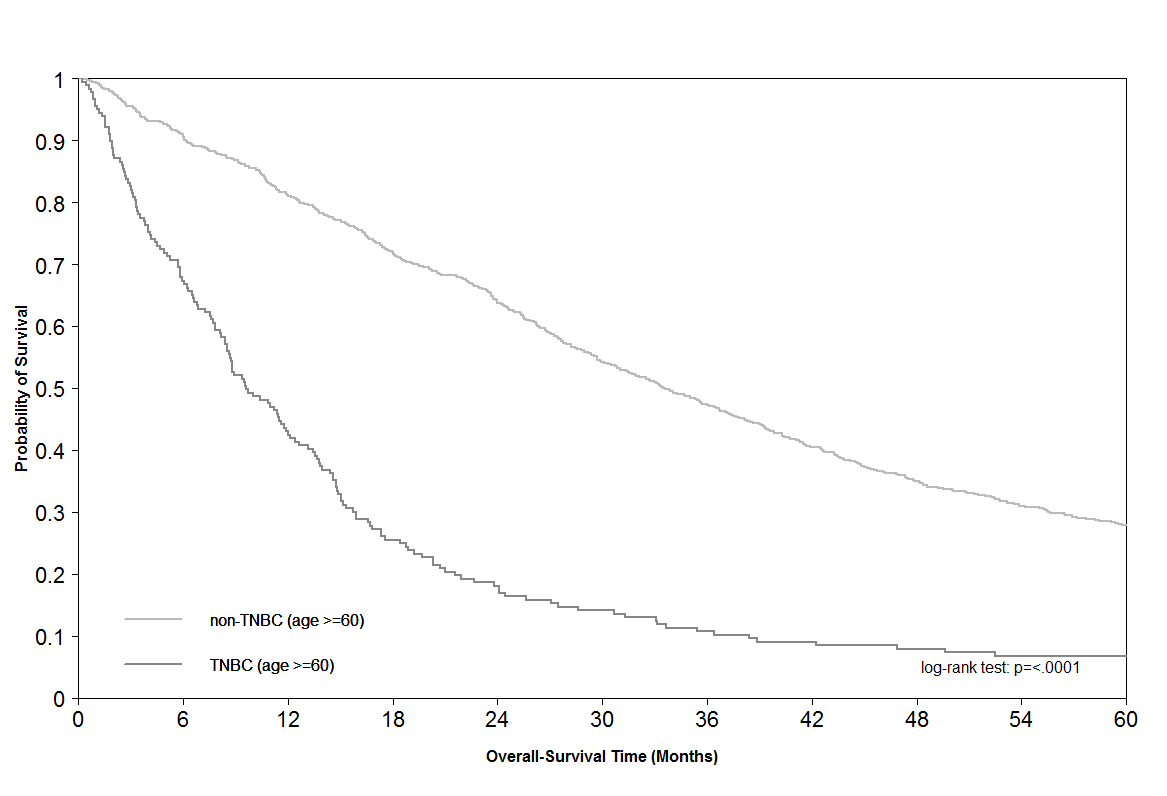


Supplementary Figure 3. Overall survival of MBC by lymph node status: (A) lymph node (-); (B) lymph node (+).

(A)


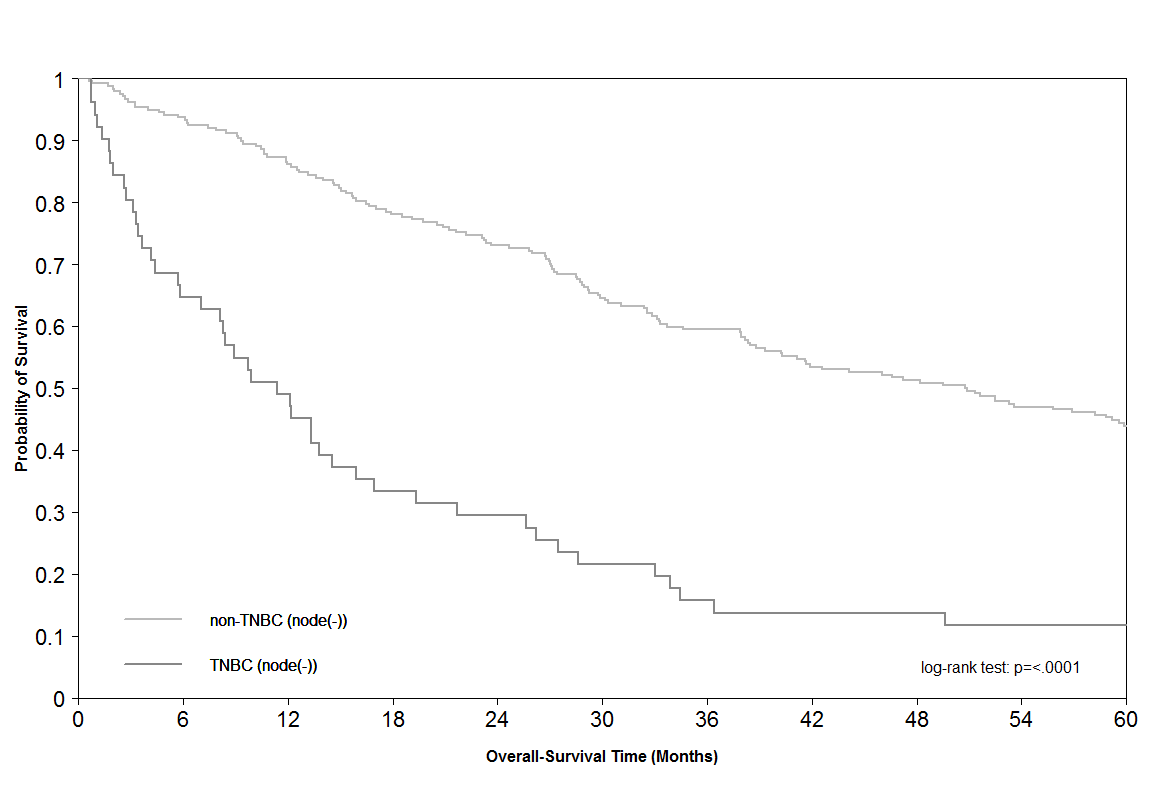


(B)


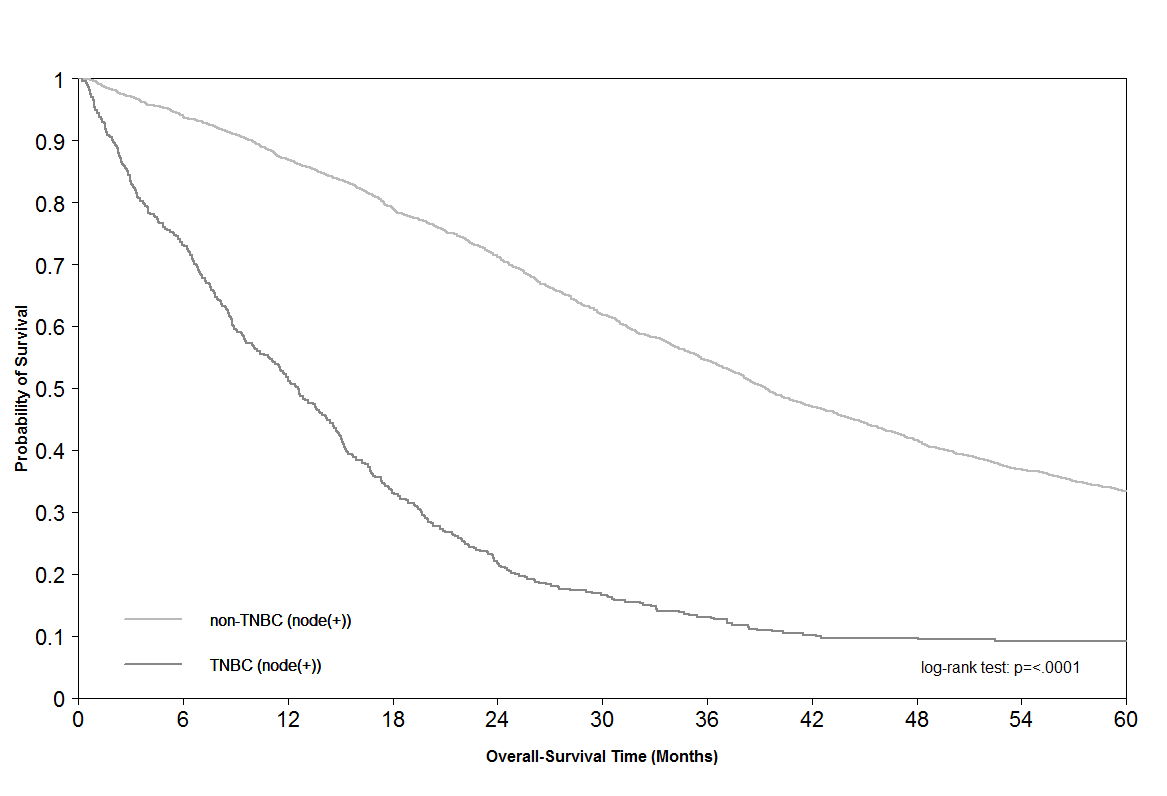


Supplementary Figure 4. Mean medical cost per patient per year after diagnosis with metastatic non-TNBC or mTNBC: (A) by age; (B) by lymph node status.

(A)


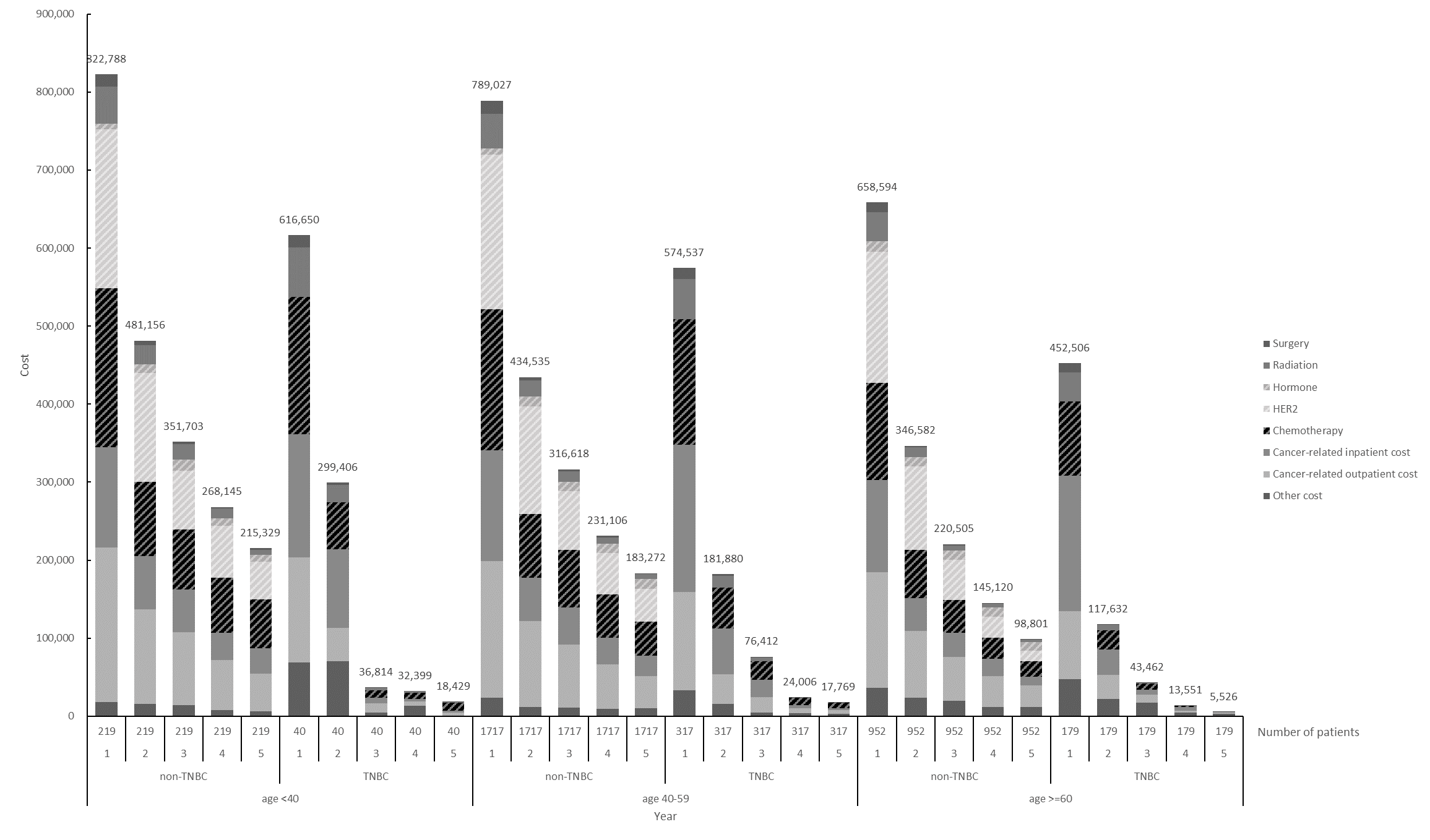


(B)


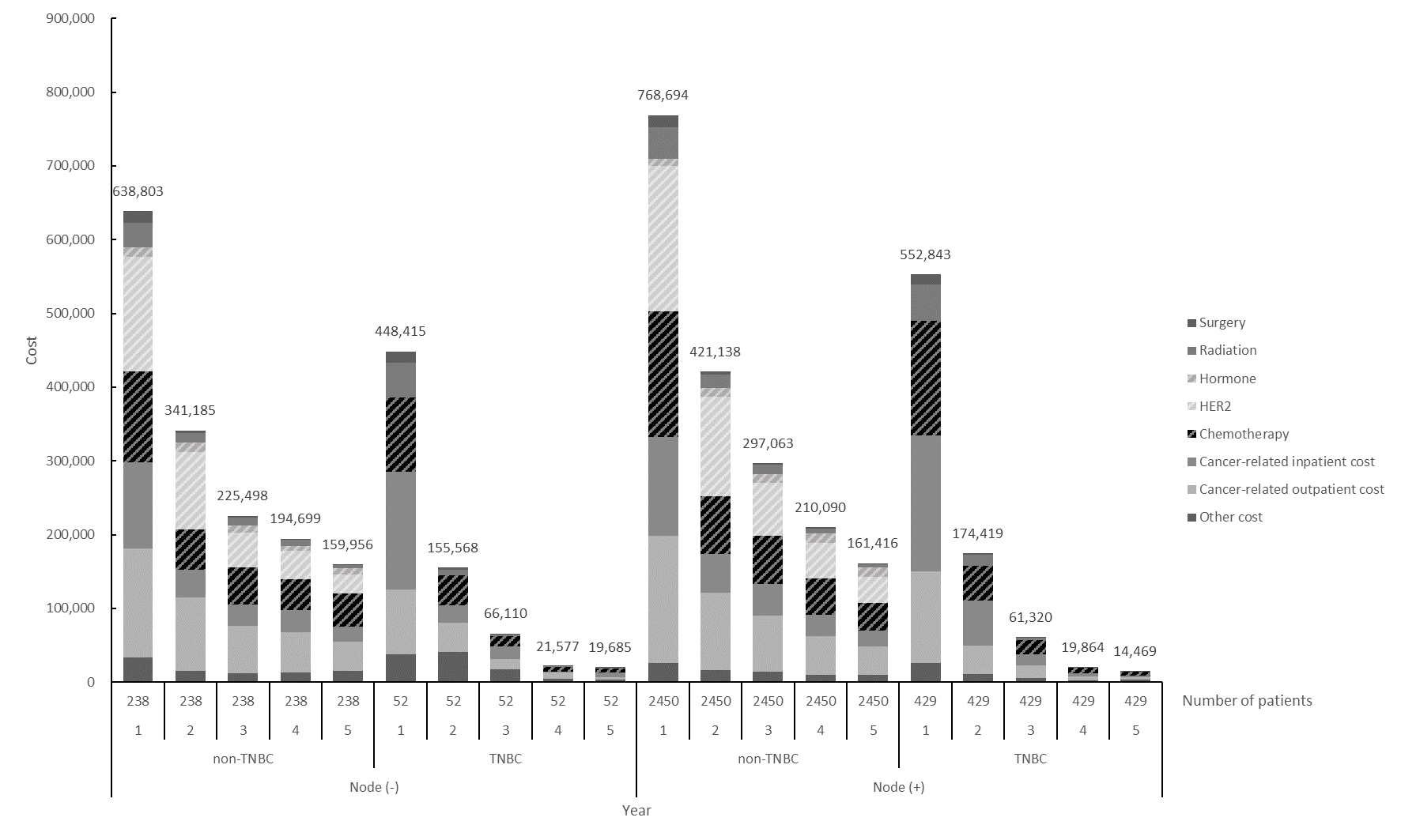

Supplement: Supplementary file 1 — Supplementary Information. [file 41598_2021_4316_MOESM1_ESM.docx]
